# Climate change and public health in California: A structured review of exposures, vulnerable populations, and adaptation measures

**DOI:** 10.1073/pnas.2310081121

**Published:** 2024-07-29

**Authors:** Michael Jerrett, Rachel Connolly, Diane A. Garcia-Gonzales, Claire Bekker, Jenny T. Nguyen, Jason Su, Yang Li, Miriam E. Marlier

**Affiliations:** ^a^Department of Environmental Health Sciences, Fielding School of Public Health, University of California Los Angeles, Los Angeles, CA90095; ^b^Department of Environmental Health Sciences, School of Public Health, University of California Berkeley, Berkeley, CA94720; ^c^Department of Environmental Science, Baylor University, Waco, TX76798

**Keywords:** climate change, public health, vulnerable populations, cobenefits

## Abstract

California faces several serious direct and indirect climate exposures that can adversely affect public health, some of which are already occurring. The public health burden now and in the future will depend on atmospheric greenhouse gas concentrations, underlying population vulnerabilities, and adaptation efforts. Here, we present a structured review of recent literature to examine the leading climate risks to public health in California, including extreme heat, extreme precipitation, wildfires, air pollution, and infectious diseases. Comparisons among different climate-health pathways are difficult due to inconsistencies in study design regarding spatial and temporal scales and health outcomes examined. We find, however, that the current public health burden likely affects thousands of Californians each year, depending on the exposure pathway and health outcome. Further, while more evidence exists for direct and indirect proximal health effects that are the focus of this review, distal pathways (e.g., impacts of drought on nutrition) are more uncertain but could add to this burden. We find that climate adaptation measures can provide significant health benefits, particularly in disadvantaged communities. We conclude with priority recommendations for future analyses and solution-driven policy actions.

## Significance

We examine the most severe climate-related exposures and health effects in California. Health effects vary by certainty, timing, potential health burden, vulnerable populations affected, and available adaptation measures. Throughout this review, we cover various adaptation measures and cobenefits that can accrue along with mitigation. We find that for several of the most certain exposures and health effects, adaptive and mitigative solutions are already available. Climate change, however, presents numerous cross-sectoral challenges that can hinder integrative public health climate solutions.

California has long been a global leader in climate mitigation policies with the adoption of the world’s first legally binding limits on carbon dioxide emissions (1). Due to California’s Mediterranean climate and additional pressures associated with resource management, however, the State’s population also experiences some of the most severe public health impacts from climate change (2). Climate change presents numerous cross-sectoral challenges that can make integrative climate solutions difficult to identify and implement. We present a structured review that allows for prioritization of health effects based on their certainty, timing, and likely burden of illness. We propose that policy responses should prioritize exposures that are most certain to result from climate change and are likely to exert substantial population health impacts. The population health model perspective underpins this proposition (3). This model has the overarching objective of investing limited societal resources in the social and environmental determinants of health most likely to maximize overall health outcomes, while reducing health inequalities at the population level (4). This Global Burden of Disease (GBD) framework also informs this proposition. Among other things, the GBD seeks to help set health priorities by understanding the magnitude of impacts across a broad spectrum of health outcomes, behaviors, and putative exposures (5). Finally, the proposition draws on the International Panel on Climate Change (IPCC) use of certainty or likelihood as a key component of expert assessment, with other aspects relating to evidence agreement and confidence in the findings. Implicitly, the IPCC seeks to identify inherent uncertainties in potential impacts of climate change, recognizing how the certainty of an outcome is one of the key criteria determining priorities in the human policy response (6).

We have organized the paper into the following sections: (1) a general framework for identifying and understanding the major health effects of climate change in California; (2) a review of key climate change exposures that affect health in California (i.e., extreme heat, extreme precipitation, wildfires, air pollution, and infectious diseases) and related adaptation measures; and (3) a synthesis of the review and suggestions for future research and solutions. We also identify and analyze health cobenefits or benefits to public health that occur as beneficial side effects of adaptation and mitigation actions.

## Framework to Evaluate Key Climate-Health Threats in California

**Fig. 1.**
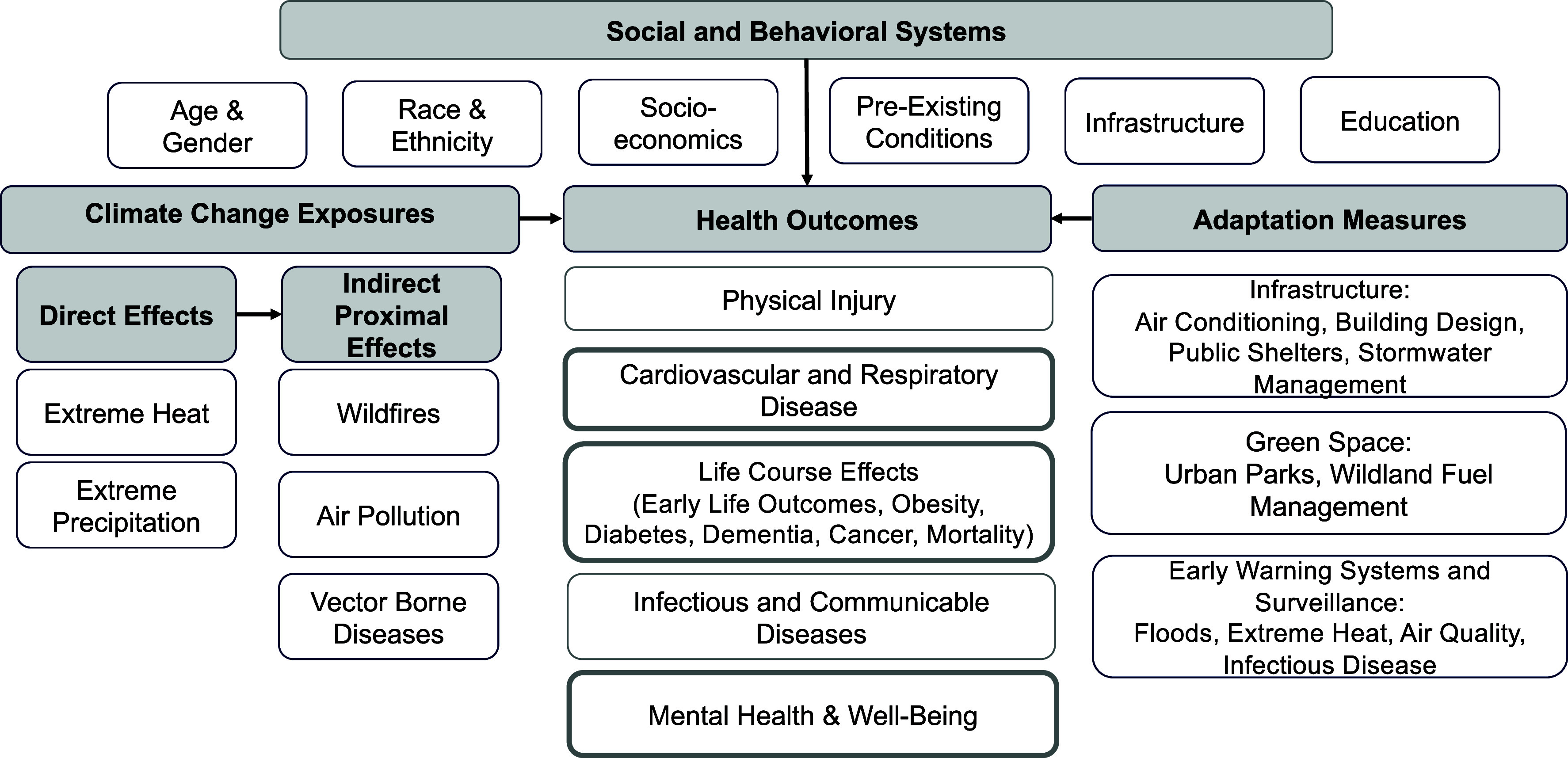
Conceptual diagram of the connections between direct and proximal indirect climate change exposures, social and behavioral systems, health outcomes, and adaptation measures. Health outcomes in thick outlined boxes (cardiovascular and respiratory disease, life course effects, and mental health) relate to the current leading causes of death and disability for California. See main text for a description of specific linkages among categories.

Here, we focus on the direct and proximal indirect climate exposures in California, including extreme heat, extreme precipitation, wildfires, air pollution sources and formation, and infectious disease (Fig. 1). To structure the review, we established a series of evaluative criteria for each selected climate risk (Table 1), including the attribution of exposure to climate change as defined by the Fifth National Climate Assessment, the projected timing of the effects, the likely burden of disease related to the exposure, vulnerable populations, and adaptation measures. We focus on California-specific studies, but also consider those from other locations when no studies from California were available or when we are seeking to link California to national or international policies. We examine health effects in the general population and in vulnerable groups. We focus on occupational health risks only when these groups are at higher risk for adverse exposures and subsequent health effects (e.g., outdoor workers and extreme heat); a full review of occupational health risks is beyond the scope of this paper. We also review West Nile Virus, aeroallergens, and air pollution formation, but because of lower attribution certainty, smaller health burden, or both, only a brief summary is provided in the main text with a full description in *SI Appendix*.

**Table 1.** Evaluation criteria to assess the direct and proximal indirect health effects of climate change in California and summary of results of structured literature review^*^

	Climate pathway		Attribution	Statewide health burden	Health outcomes	Vulnerability	Adaptation
			What is the confidence of exposure attribution to climate change?	What is the magnitude of the health burden?	What health outcomes are impacted?	Are certain populations more vulnerable to health effects?	What adaptation measures are available in California?
Direct	Extreme heat		High^†^	• 10 s of deaths, 1,000 s hospitalizations, 10,000 s excess ER visits for 2006 heatwave event • 10 s of adult deaths for each extreme heat day	• Cardiovascular and respiratory disease • Life course effects • Mental health and well-being	• Age & Gender: Children; Elderly; Pregnant women • Preexisting conditions • Low SES • Education • Outdoor workers	• Infrastructure: Building design; Reflective surfaces; Air conditioning • Early warning systems: Heat warning; Heat action plans • Green space: Urban vegetation
	Extreme precipitation		High^‡^	• Unknown	• Physical injury • Mental health and well-being	• Low SES • Racial and ethnic minorities • Education	• Infrastructure: gray stormwater management • Early warning systems: Evacuation plans • Green space: Green stormwater management
Indirect proximal	Wildfires		High	• 100 s of deaths (1992 to 2022)	• Physical injury • Mental health and well-being	• Age & Gender: Children; Elderly • Preexisting conditions • Education	• Green space: Wildland fuel management; Defensible space near built structures
	Air pollution	Wildfire smoke (Source)	High^§^	• 10,000 s deaths (2008 to 2018)	• Cardiovascular and respiratory diseases • Life course effects	• Age & Gender: Children; Elderly; Pregnant women • Preexisting conditions • Low SES • Racial and ethnic minorities	• Infrastructure: Clean air shelters • Early warning systems: Air quality alerts; Personal exposure tracking
		Dust (Source)	High^§^	• 100 s deaths^¶^	• Cardiovascular and respiratory diseases • Life course effects	• Age & Gender: Children; Elderly • Outdoor workers	• Green space: Vegetation management
		Aeroallergens (Source)	Medium^#^	• Unknown	• Cardiovascular and respiratory diseases	• Preexisting conditions	
		Formation	Not formally assessed in NCA	• 10,000 s deaths for future climate scenarios	• Life course effects • Cardiovascular and respiratory diseases	• Age & Gender: Elderly; Women • Preexisting conditions • Low SES	
	Valley fever		Not formally assessed in NCA	• 1,000 s of cases for 2019 • 10 s of annual deaths and 1,000 s annual hospitalizations for 2015 to 2021	• Infectious and communicable diseases • Cardiovascular and respiratory diseases	• Age & Gender: Elderly; Pregnant women • Preexisting conditions • Racial and ethnic minorities	• Early warning systems • Green space: Vegetation management
	West Nile virus		Not formally assessed in NCA	• 1,000 s cases and 100 s deaths (2003 to 2022)	• Infectious and communicable diseases • Life course effects	• Elderly; Preexisting conditions; Outdoor workers	• Early warning systems: Surveillance

SES refers to socioeconomic status; ER refers to emergency room visits; NCA refers to the National Climate Assessment.

^*^Please refer to the main text for citations.

^†^Fifth National Climate Assessment: Climate trends for the western U.S. (Chapter 2) and Southwest region (Chapter 28).

^‡^Fifth National Climate Assessment: Southwest region (Chapter 28).

^§^Fifth National Climate Assessment: U.S.-wide (Chapter 14).

^¶^U.S. Southwest (per year) for 1996 to 2015.

^#^The Fifth National Climate Assessment indicates high confidence for some regions (Chapter 14), but California-specific studies (cited in the main text) suggest additional uncertainty, so an attribution level of medium confidence is assigned here.

Understanding public health risks to Californians requires knowledge of the leading causes of disability and death. Direct and indirect climate change exposures that can affect several highly ranked causes of disability and death will often have substantial health impacts because of the larger populations affected. The top three causes of death and disability in California primarily result from chronic disease conditions, including ischemic heart disease, stroke, and chronic obstructive pulmonary disease (*SI Appendix*, Fig. S1 ) (7). Disability adjusted life years, a combined measure of death and disability, includes ischemic heart disease, low back pain, and drug-use disorders as the top three positions. Several of the leading causes of death and disability have associations with climate change exposures.

We have excluded from this review more distal risks such as the impact of drought on water and food supplies because the downstream effects on public health are less certain than the direct and proximal indirect effects. For example, if drought were to reduce agricultural output in California, this may or may not influence the food intake of individuals because substitution of out-of-state imports could augment food supplies or California could invest in innovative solutions for reducing water use in agriculture (8). Thus, the impact of drought on nutritional intake remains uncertain. In contrast, direct heat effects and indirect proximal exposures such as wildfires exert certain, quantifiable impacts on human health through well-established biological pathways.

Some populations experience higher vulnerability to climate change exposures due to differential and cumulative exposures; lack of adaptation measures, resources, and capacity; and higher baseline levels of disease and disability (9). When discussing specific groups such as the elderly or children, we refer to “vulnerable populations,” whereas when discussing certain geographic areas we refer to “disadvantaged communities” (DACs) as defined in State legislation SB 535. The DAC designation indicates a combined disadvantage in the sociodemographic composition, health conditions such as asthma, and environmental exposures in an area, as quantified by the CalEnviroScreen tool (10). Individuals in these groups and places may suffer worse effects than the general population, which may require special attention in public health policies, and they are sometimes afforded higher levels of protection in environmental regulation and associated public health policies.

In the next section, we review the key climate threats to public health in California, following the criteria laid out in Table 1.

## Key Climate Exposure and Health Pathways in California

### Extreme Heat.

Climate change is increasing the frequency and intensity of extreme heat events. The relationship between extreme heat and health is complex, with many factors influencing the magnitude and nature of associated health impacts (11). Although no standard definition of an extreme heat event exists, increasing heat from climate change poses a substantial threat to public health (12). High confidence exists in the attribution of extreme heat to climate change; these changes are already occurring and are expected to increase in the future (Table 1 and *SI Appendix*).

#### Likely population health burden.

Extreme heat is the leading weather-related cause of mortality in the United States (13). The burden of extreme heat on human health in California is already substantial and expected to increase; the Fifth National Climate Assessment states with high confidence that increases in extreme heat are adversely impacting human health in the U.S. Southwest (14). Extreme heat can raise body temperature and result in increased breathing rates, elevated heart rates, and changes in blood coagulation and arterial pressures. These disruptions in key circulatory mechanisms can lead to adverse cardiac outcomes because of the added demand induced by extreme heat exposures. Considerable evidence shows that extreme heat can exacerbate chronic conditions, increase the risk of heat-related illnesses, and lead to multiple morbidities including cardiovascular, respiratory, renal, and, in severe cases, death (15–17). Extreme heat has also been associated with negative impacts on pregnancy, mental health outcomes, and an increased risk for hospitalizations and emergency room (ER) visits, especially in vulnerable populations (17–19).

Limited information exists on the overall health burden from extreme heat within California; however, a study examining all-cause mortality within the contiguous United States found that each additional extreme heat day was associated with 0.07 additional deaths per 100,000 adults (20). With California’s roughly 30 million adult residents, this would correspond to 21 deaths across the state’s adult population for each extreme heat day. Between 2008 and 2017, the median number of extreme heat days during the summer months, measured across all 3,108 counties within the contiguous United States, was 89 d (20). California-specific climate models indicate that by the end of the century, under RCP 8.5 scenario, the average number of extreme heat days could rise to 52 annually across all counties (21). This average varies by county, with some areas potentially experiencing more or fewer heat days. Using the current state population, this increase could translate to approximately 1,100 heat-related deaths annually across California, assuming no changes in population vulnerability or adaptive capacity. Analysis of historic heat events provides additional evidence. The July 2006 heat wave affected much of California and was characterized by nearly 2 wk of triple-digit temperatures, higher-than-normal humidity, and high nighttime temperatures. This resulted in an estimated 232 deaths across nine counties, increasing all-cause mortality risks by 9% for each 10°F increase in apparent temperature (22). When we consider morbidity impacts, the population burden rises even further. During the same 2006 heat wave, a total of 16,166 excess ER visits and 1,182 excess hospitalizations occurred statewide (15). In the absence of adaptation measures, the impact of extreme heat will rise by the end of the century, with models predicting approximately 400 deaths per one million persons every year (23).

#### Vulnerable populations.

High-risk populations include children and the elderly; pregnant women; individuals living in DACs; undocumented, economically disadvantaged, and unhoused individuals; individuals with preexisting health conditions; and outdoor workers (15, 24–26). Children, the elderly, and those with preexisting health conditions are less likely to self-thermoregulate and can suffer from impaired thirst sensation or experience impaired glomerular filtration rates. Additional studies examining the impacts of extreme heat on mortality found that the Black racial/ethnic group had a higher risk of nonaccidental mortality with 10°F increases in temperature compared with Whites (16); however, racial susceptibilities to heat-related health impacts varied across studies suggesting modification by additional factors such as medication use and comorbidities (27). In addition, extreme heat exacerbates existing inequities across the State by impacting DACs that are disproportionately located within some of the hottest census tracts in the state (28), in addition to having economic barriers to air conditioning (AC) use (29). Vegetation in DACs tends to be less drought resistant and more sensitive to meteorological drought conditions; thus, DACs are less likely to benefit from urban green space cooling effects during hot and dry conditions (30). These conditions are likely due, in part, to barriers to water access and usage, which may result in a positive feedback loop between extreme heat and declining vegetation.

#### Adaptation measures.

For indoor environments, residential AC has dramatically reduced extreme heat-related mortality since the 1960s (31) and heat-related illnesses in California (32). Access to AC is a challenge because penetration across California (33, 34) and the United States is inequitable, with less access for low-income and racially-minoritized populations (29, 35, 36). Individual perceptions of heat exposure risks and the cost of operating AC may impact personal decisions on AC use (27, 37). Furthermore, AC uses electricity often generated from fossil fuels, exacerbating greenhouse gas emissions (27).

Urban green spaces, specifically tree canopies, have been widely recognized for their role in reducing urban heat islands via evapotranspiration and shading from solar radiation (38–41). Within Los Angeles, achieving moderate tree cover and increased albedo reflectivity (“white-scaping”) could delay climate-induced warming by approximately 70 y; in effect, experiencing a climate in the year 2089 that was like the climate in the year 2020 (42). Aggressive action to increase both tree canopy and urban reflectivity within urban Los Angeles could save one in four lives during extreme heat events (42); however, it is important to plant drought-tolerant vegetation to decrease inequities in water access and usage, and increase resiliency to local drought conditions, as noted above (30, 43). Cool roofs, designed to reflect more sunlight and absorb less heat relative to standard roofs, can also reduce urban temperatures (44). Cool roofs have been predicted to reduce future exposure to heat waves across the 29 most populous counties in California by up to 56% (45). The combination of cool and green roofs with street trees can decrease both projected regional and summer temperatures across the United States with the highest impacts in the Southwest (44).

Additional adaptive solutions include well-communicated local-level heat action plans, robust health surveillance and monitoring programs, cooled public spaces, and interventions aimed at the most vulnerable populations such as low-income elderly people who may lack both AC and a social network to monitor their health. Targeting adaptive actions within areas with limited access to either private or public cooled spaces could reduce heat-related health risks (46). For example, shared-wall, multifamily dwelling units can reduce peak energy demand by up to 50%, as observed in Los Angeles (47); thus, increasing shared-wall housing stock has the potential to decrease regional energy demands and reduce costs associated with energy usage for residents. Furthermore, California-based tools like the California Healthy Places Index: Extreme Heat Edition (48) and the Center for Healthy Climate Solutions Heat Maps (49) help users visualize and understand the distribution of heat-related vulnerabilities and illness across the state, identify adaptation resources, and to prioritize the delivery of resources and programs (49, 50). Combining heat mitigation and adaptive measures can decrease urban temperatures and adverse health outcomes more than any single management action (51).

### Extreme Precipitation.

Climate change alters the timing and severity of extreme precipitation events. In 2023, several locations in California experienced heavy rain, extreme winds, flooding, and landslides associated with Hurricane Hilary, the first tropical storm to hit the state since 1939 (52). Here, we review public health risks associated with wet precipitation extremes. Selected health burdens associated with droughts are explored in subsequent sections. High confidence exists in the attribution of extreme precipitation to climate change, the effects of which are expected to increase in coming decades (Table 1 and *SI Appendix*).

#### Likely population health burden.

Extreme precipitation events impact health through multiple pathways: trauma or drowning, displacement, water quality, vector-borne diseases, and mental health impacts (53). The Fifth National Climate Assessment assigns a high confidence designation to adverse physical health impacts from flooding in the Southwest region (14). If a hypothetical severe winter storm of a comparable magnitude to the historic 1861 to 1862 events in California were to occur today, the State would face widespread flooding, extreme winds, and landslides. Given the location of development and residential areas, this hypothetical event could cause hundreds of billions of dollars in property damage, the evacuation of approximately 1.5 million residents, and disruption to critical infrastructure, particularly among Central Valley and coastal communities (54). More concretely, 1.09 million properties in California are currently at risk of flooding, which could increase to 1.16 million in the next 30 y (55, 56). Water management planning is and will continue to be challenged by the shifting nature of precipitation events (57, 58).

Stormwater runoff also accumulates pollutants that can negatively impact water quality. Intense precipitation during atmospheric river events, for example, is linked to three-quarters of fecal pollution spikes in California’s coastal waters, including in densely populated areas in Southern California (59). Drier summers could possibly reduce coastal water contamination through reduced runoff amounts, but increased population density in coastal areas and increased variability in precipitation could challenge surveillance used to mitigate health risks (60). Precipitation extremes could also amplify the effects of climate-related sea level rise: coastal flooding, erosion, salinization of water sources, and storm surges (61). In coastal California, hazardous sites are vulnerable to flooding, which can release toxic chemicals into the environment. This exposure risk, particularly in DACs, is expected to increase by the end of the century (62). Currently, however, existing literature lacks comprehensive investigations of the past, current, or future burden of morbidity and mortality from extreme precipitation events.

#### Vulnerable populations.

Populations vulnerable to extreme precipitation include DACs and people who have inequities in access to information to prepare for extreme events or have increased incidence of chronic illnesses or other impairments that can reduce evacuation ability. A recent study in Los Angeles found that approximately 425,000 people are at high risk of flooding, with DACs and Black communities being disproportionately impacted (63). Communities with limited English proficiency are at risk for timely evacuation and access to flood alerts (64). Certain populations will be more vulnerable to water quality changes, including children (65).

#### Adaptation measures.

Retreating from flood-prone areas or prohibiting development in flood zones can limit future populations at risk of flooding. Efforts to improve wastewater, drainage, and flood management, as seen in San Francisco, could also reduce risk (66). Impervious surfaces increase the risk of flooding associated with climate change in California (67). Tree canopies or other green infrastructure can reduce the volume and intensity of stormwater runoff in urban areas (68, 69). In addition, prediction of extreme events, including monitoring, flood alerts, and evacuation plans, can protect public health.

### Wildfires.

Wildfires have direct impacts on human health, through the loss of life and physical injuries, and more indirect impacts on mental health and the ability to meet health care needs. High confidence exists in the attribution of wildfires to climate change, the effects of which are expected to increase in coming decades (Table 1 and *SI Appendix*). We review the wildfire contribution to air pollution in a subsequent section.

#### Likely population health burden.

The Fifth National Climate Assessment assigns a high confidence designation to adverse physical health impacts from wildfire activity in the Southwest region (14). According to CAL FIRE, from 1992 to 2022, wildfires killed 268 civilians and firefighters (70). Emerging research suggests mental health effects as well. Following the 2018 Camp Fire, the deadliest wildfire in California history with a death toll of 85 civilians, direct exposure to the wildfire significantly increased the risk for mental health disorders, especially posttraumatic stress disorder (PTSD) and depression (71). Individuals seeking emergency relief services after a wildfire were at an increased risk for psychopathology, such as symptoms of major depression and PTSD (72). Evacuations during wildfires can also impact health care needs and the ability to meet those needs (73).

#### Vulnerable populations.

Firefighters are critically exposed to the risks of wildfire-related injury, mortality, and mental health effects. Since 2008, 32 firefighters in California have died fighting wildfires (70). CAL FIRE also reported increasing reports of suicide, grief, and addiction or substance abuse among firefighters coinciding with increasing wildfire activity, and several studies have found increased incidence of PTSD, major depressive disorder, and anxiety disorders in the general population following large-scale wildfires (70, 74, 75).

Elderly people and people with disabilities are often more vulnerable to the physical and mental health effects of wildfires because they face additional barriers during evacuations. Children and youth with disabilities face unique mental health impacts from wildfire-related displacement. Children and their parents exhibited stress, grief, and other emotional and behavioral reactions during evacuation and up to a year post-wildfire exposure in northern California (76).

People with chronic health conditions can also be more vulnerable during wildfires and evacuations. Wildfires can affect health care utilization for people using electricity-dependent medical devices. During the southern California wildfires in 2003, increased wildfire activity and pollution was associated with a reduced rate of all-cause outpatient visits 1 d after exposure and increased health care utilization 4 to 5 d after (77). Their results suggest that wildfire exposure interrupts routine care for people who use electricity-dependent medical equipment, but also results in increased health care utilization following wildfires. Ultimately, protections for at-risk communities will require multidisciplinary and holistic approaches to managing wildfire threats, as well as fostering community resilience and adaptive capacity (78).

#### Adaptation measures.

Green space management in agricultural areas, parks, recreation areas, or other managed open spaces in the wildland–urban interface can reduce wildfire risk in California (79). Buffering the landscape with less flammable land uses and fuel reduction can reduce wildfire spread and severity (79, 80). Moritz et al. emphasize the need for coordination among landscape-scale interventions with the built environment (e.g., fire-resistant construction) and across the community scale (e.g., community outreach to create social capital that increases mitigation efforts) (79). Increasing defensible space reduces available fuel in proximity to homes and can minimize the risk of homes igniting, as observed in San Diego (81). The relationship between vegetation and wildfire risk (as measured through structure loss), however, is complex and varies from local to landscape scales (82).

### Air Pollution.

Poor air quality has long been a leading public health issue in California, with significant implications for population health burdens. Recent studies have estimated up to 40,400 deaths per year could be attributed to PM_2.5_ and O_3_ exposure (83). Climate change can affect air pollution both through the contribution to emissions sources and the formation of secondary pollutants in the atmosphere. The Fifth National Climate Assessment assigns medium confidence to overall climate-attributable adverse air quality impacts across the United States (84). We first review the public health consequences of climate-sensitive emissions sources, focusing on wildfires and dust in the main text. Because of relatively lower certainty, aeroallergens and the effects on physical and chemical processes for air pollution formation are in *SI Appendix*.

#### Climate-Sensitive Air Pollution Sources (Wildfires).

Climate change influences wildfire smoke pollution through increased and more severe fire activity, which results in higher emissions. Wildfires contribute to air pollution, including fine particulate matter (PM_2.5_), ozone (O_3_), nitrogen dioxide (NO_2_), and other trace gases. Wildfires have also become an increasingly important source of carbon dioxide emissions (85). In this section, we review the health implications of wildfire smoke pollution. There is high confidence in the attribution of wildfire smoke pollution to climate change, the effects of which are expected to increase in coming decades (*SI Appendix*).

##### Likely population health burden.

Numerous studies have documented the detrimental health effects of wildfire smoke exposure. Exposure to wildfire smoke PM contributes to oxidative stress, inflammation, and cell toxicity (86), and studies have found that wildfire smoke PM has greater oxidative potential than ambient urban PM, likely resulting in greater toxicity (87, 88). Wildfire smoke also increases indoor PM_2.5_ exposure in California where mean indoor concentrations of PM_2.5_ nearly tripled during wildfire events (89); this is an important contributor to health effects given that people spend the majority of their time indoors (90). Wildfire smoke exposure is consistently associated with increased respiratory morbidity and all-cause mortality (91–93). In studies focused on California, researchers found positive associations between smoke exposure and ER visits and hospitalizations for respiratory symptoms among the general population and especially asthmatics (94–98). Recent evidence from Southern California wildfires also suggests that wildfire smoke pollution may be more harmful to respiratory health than other pollution sources (99). The effect of wildfire smoke exposure on cardiovascular health is less conclusive. Some studies, including those from California, did not find an association for cardiovascular events or cardiovascular-related mortality (97, 100). Recent synthesized evidence, however, shows that wildfire PM exposure is a risk factor for cardiovascular morbidity and mortality (101). In their review, Chen et al. (101) found that 25 out of 38 epidemiological studies on cardiovascular morbidity found a positive association between wildfire smoke exposure and cardiovascular disease impacts. Emerging evidence links wildfire exposure to adverse birth outcomes, such as preterm births, via smoke exposure or psychosocial stress pathways, but this requires further study (102, 103).

Several studies have estimated annual mortality attributable to smoke PM_2.5_ exposure for the western United States and contiguous United States. For the contiguous United States, estimates range from the more conservative O’Dell et al. estimate of 6,300 deaths (CI: 4,800 to 7,200) to the Fann et al. range of 8,700 to 32,000 deaths attributable to smoke PM_2.5_ exposure, depending on the time period and dose–response function applied (104, 105). A recent study by Wang et al. (106), which addressed the impact of the severe 2018 California wildfires, found that smoke pollution contributed to over 3,600 statewide deaths that year. The total wildfire damages amounted to $148 billion in health costs, capital, and other indirect losses (106). Connolly et al. quantified the mortality burden for fire PM_2.5_ exposure in California over a longer time period, 2008 to 2018, and found that 52,480 to 55,710 premature deaths are attributable to wildland fire PM_2.5_ over the 11-y period, with an estimated economic impact of $432 to $456 billion (107). With respect to predictions of future smoke exposure-related mortality, limited information exists on the burden for California specifically. Focusing on future western U.S. wildfire activity, Neumann et al. project that wildfire PM_2.5_-related mortality could increase by 3.7 to 4.2 times by the end of the century under moderate (RCP 4.5) and high (RCP 8.5) emissions scenarios respectively (108). Climate change accounts for 40% (for RCP 4.5) to 60% (for RCP 8.5) of the projected increases in wildfire mortality (108).

##### Vulnerable populations.

The sensitive groups for wildfire smoke PM_2.5_ exposure—people who are at greater risk of experiencing harmful effects—include children younger than 18 (especially children under the age of 5), adults 65 or older, people with chronic health conditions such as asthma, DACs, and potentially women and racially-minoritized populations. Most of the literature focuses on differential impacts by age group; children, especially under 5 y of age, are more susceptible to the respiratory health effects of wildfire smoke pollution, including ER visits for respiratory symptoms, asthma diagnoses, and asthma hospital admissions (94, 95). Aguilera et al. also found that wildfire-specific PM_2.5_ was 10 times more harmful to children’s respiratory health compared to other PM_2.5_ sources for emergency and urgent care from 2011 to 2017 in San Diego (109). Older populations over age 65 are often more susceptible to respiratory and potentially cardiovascular impacts from smoke pollution exposure (94, 98). Recent research in California has reported that higher smoking prevalence rates modify the effect of wildfire smoke on ER visits for asthma and pneumonia, with greater admissions in areas with higher smoking rates (110).

Current evidence is mixed on the extent to which low-income populations and racially-minoritized communities are differentially exposed to air pollution from wildfires (111–113), though recent evidence from California indicates that wildfire smoke exposures may exacerbate existing health disparities and add to a cumulative environmental burden in DACs (114); evidence of differential susceptibility to health impacts from smoke in low-income populations, elderly women and Black populations remains limited (97). A community health vulnerability index demonstrates that U.S. counties with higher levels of wildfire-associated PM_2.5_ also have higher levels of sensitivity (with respect to age, occupation, and preexisting conditions) and lower adaptive capacity (115).

##### Adaptation measures.

Adaptation measures for wildfire smoke will involve a variety of tools, including public health messaging and air filtration technology. In California, public health messaging that incorporates simple, consistent language was more commonly remembered, understood, and complied with; however, multiple modes of communication can be useful to reach a wider audience (116). Providing clean air shelters and portable air cleaners may also reduce individual exposure (117).

Fuel management strategies, such as prescribed burning, can potentially reduce the severity of wildfire pollution, reduce carbon emissions, and mitigate climate change with long-term health benefits. If widespread prescribed burning were to be implemented to reduce wildfire activity in the western United States, biomass-burning carbon emissions could be potentially reduced by up to 25% (118). Simulations of land management policy scenarios in California have studied the net impacts of land conservation, ecosystem restoration, forest fuel reduction (i.e., prescribed burning and mechanical thinning), and agricultural carbon sequestration on carbon dioxide and methane emissions (119). Although emissions from prescribed burning initially outweighed emissions reductions from other strategies, cumulative net emissions would be reduced by midcentury and more significantly by the end of the 21st century. The quantifiable net impacts of prescribed burns on carbon dioxide and particulate emissions, however, remain uncertain, because research has yet to ascertain accurately the extent to which low-level prescribed burns will prevent future emissions from extreme wildfires (120).

Important tradeoffs exist between the air quality and public health costs of prescribed burning and wildfires. In California, high-intensity wildfires expose larger regions to more intense smoke pollution, while lower-intensity prescribed burns result in more localized pollution and certain communities may be disproportionately exposed (121). Overall, research suggests that wildfires result in greater smoke pollution concentrations and exposure than prescribed burns. A recent paper modeled air quality impacts in California from past wildfires and prescribed burns (2008 to 2017) and estimated how more than doubling annual prescribed burn area (to >200,000 ha/y) would impact air quality over the course of 8 y. They found that smoke concentrations from the target prescribed burning scenarios were lower than the concentrations from baseline wildfires and prescribed fires in 95.2% of California ZIP codes (122).

#### Landscape Dust (Pollution Source).

The arid and semiarid region of the southwestern United States is characterized by large concentrations of soil-derived dust particles in the lower atmosphere, especially in spring. There is high confidence in the attribution of dust pollution sources to climate change, the effects of which are expected to increase in coming decades (Table 1 and *SI Appendix*).

##### Likely population health burden.

The size of dust particles typically spans from less than 1 μm to 400 μm in diameter, with particles larger than 100 μm mostly settling down near the source of formation. Particles less than 10 μm (PM_10_) can reach the thorax into the human lung, with fine dust particles (e.g., PM_2.5_) posing the greatest risk to health because they can reach the alveoli and affect the oxygen-blood exchange, thus having been the most closely studied (123). Distinct dust constituents such as crystalline silica and endotoxins have long been studied and are found to be highly associated with adverse respiratory outcomes, including a decline in lung function, pulmonary diseases, lung cancer, and silicosis (124, 125). Dust may also transport harmful neurotoxic pesticides that can come into contact with sensitive human receptors such as children, with possible impacts on neurological development (126). Exposure of farmers in California to endotoxin, a type of bioaerosol, as well as crystalline silica, has also been associated with allergy, respiratory, and lung diseases (127–129). Across the U.S. Southwest, the present-day health burden of dust PM_2.5_, relative to a counterfactual of zero, is 590 deaths per year for all adults, along with 160 and 130 hospital admissions for cardiovascular and respiratory disease for older adults (130). Achakulwisut et al. estimated that the all-cause mortality burden from dust in the U.S. Southwest could increase 230% and 210% by the end of the century under RCP 8.5 for coarse (PM_10_) and fine (PM_2.5_) particulate pollution, respectively (131).

##### Vulnerable populations.

Larger particles are not transported as far in the atmosphere; however, many rural communities, including in the San Joaquin Valley, will be vulnerable to dust pollution exposure (132). Adverse birth outcomes (low birth weight and prematurity) have been linked to dust storms in the United States (133). Children and the elderly are projected to be more vulnerable to cardiorespiratory mortality and morbidity attributed to climate-induced changes in coarse and fine dust pollution in the U.S. Southwest (131). Outdoor workers in agriculture and construction face growing threats from dust exposure, particularly from Valley Fever (see the *Infectious Disease* section below) (134).

##### Adaptation measures.

Developing early dust warning advisory and assessment systems, reducing personal exposure by reducing time spent outdoors and engaging in outdoor physical activities during dust events, and using devices such as wearable GPS and activity sensors to track individual locations and activities—and give customized warnings and suggestions—could also assist with the mitigation of dust-related health outcomes (135). In certain areas of California such as the San Joaquin Valley, agricultural activities and wind erosion drive landscape dust sources (132). As a result, green space expansion in California could mitigate dust mobilization and its health consequences. Wind speed and vegetation cover are two key factors that determine soil erodibility and dust emissions (136). In addition, vegetation constrains dust emissions by preserving soil moisture through plant shade and root systems (137). Under future climate scenarios, regions with higher temperatures, reduced soil moisture, and enhanced anthropogenic land use practices could experience heightened dust mobilization (138), as the loss of vegetative cover during drought increases soil erosion (139, 140).

#### Air Pollution Formation.

Close connections of atmospheric chemical processes with atmospheric conditions have prompted concerns about the effects of climate change on air pollution formation (83). These connections are highly uncertain and were not formally assessed by the Fifth National Climate Assessment. Thus, we review the relationship between climate change and air pollution formation and the potential health burdens from specific air pollutants in *SI Appendix*.

#### Infectious Disease.

More than half of all infectious diseases are known to be influenced by climatic conditions (141). Climate change can influence the prevalence and incidence of infectious diseases via myriad exposure pathways illustrated in *SI Appendix*, Fig. S2 . For example, extreme weather can affect distributions of vectors such as mosquitoes and liberate fungal pathogens. Animal and vector ecology can also be influenced by changes in climate. Some vector-borne and fungal diseases can be influenced by rising temperatures, while water quality and quantity changes can impact several waterborne diseases.

As noted above, however, infectious diseases in general rank as relatively lesser threats to health in California than noncommunicable chronic diseases and conditions (*SI Appendix*, Fig. S1 ). The possibility exists, however, that a newly emerging infectious disease, such as SARs CoV-2 (the cause of COVID-19), can extract a huge toll on population health, and some evidence suggests that climate change can increase the risk of emerging novel pathogens (141), although no evidence suggests that climate change contributed directly to the COVID-19 pandemic. Nevertheless, such diseases cannot be discounted with respect to the huge risk they can pose; for example, as of November 12, 2023, nearly 105,000 COVID-19 deaths had been recorded in California since the start of the pandemic in 2020 (142). Some studies have reported that cooler, drier conditions may contribute to more severe COVID-19 infections; thus the link to climate change remains unclear with this virus (143). In general, however, many of the new endemic or growing infectious disease threats exert a relatively small impact on population health compared to noncommunicable diseases. In California and other parts of the southwest, climate-sensitive infectious diseases are caused by mosquitoes, ticks, and fungal infections (144). Reviewing the factors shown in *SI Appendix*, Fig. S2 goes beyond the scope of this review; thus, we focus on a few of the more important infectious diseases for California in terms of incidence, death, and sensitivity to climate change. Specifically, we focus on two diseases presenting significant public health risks: West Nile Virus (*Flaviviridae*) (145) and coccidioidomycosis or Valley fever (146). Valley fever affects more people than West Nile Virus in terms of annual incidence (8,030 versus 149 new cases in 2021) (147). A further discussion of the West Nile Virus burden is available in *SI Appendix*.

##### Valley Fever.

Valley fever is a growing concern associated with dust mobilization in the southwestern United States. Valley fever is caused by the inhalation of dust-carried fungi *Coccidioides immitis* and *Coccidioides posadasii* (148). It is endemic to California’s Central Valley. The confidence level for the attribution of Valley fever to climate change is not formally assessed in the Fifth National Climate Assessment, though effects are expected to increase in coming decades (Table 1 and *SI Appendix*).

###### Likely population health burden.

Valley fever can infect the lung system, cause respiratory symptoms such as cough, fever, shortness of breath, and chest pain, may spread to other parts of the body, and can be potentially fatal (149). In California, the number of reported Valley fever cases has greatly increased from 719 in 1998 to 9,004 in 2019 (149). On average in California, there were 80 annual deaths and 1,000 annual hospitalizations from the disease from 2015 to 2021 (150). Originally, high Valley fever incidence in California was reported in the San Joaquin Valley, but the largest case increases in recent decades have occurred in the Northern San Joaquin Valley, the Central Coast, and South Coast regions (151). Correlated changes in Valley fever incidence with drought and temperature in California demonstrate that climate conditions are driving factors that control Valley fever outbreak, leading to the expansion of Valley fever cases to regions that experience prolonged dryness and drought in recent years and under future climate scenarios (152–154).

##### Vulnerable populations.

Most cases have been contracted occupationally in construction, agricultural, military, archaeological, and correctional institutions, possibly due to heightened exposure to dust. People over 60 y of age, pregnant women, those with depressed immune systems, women, and Black and Filipino populations are at higher risk of contracting coccidioidomycosis (155–157).

##### Adaptation measures.

Matlock et al. recommend effective public health messaging through simple language across various outlets with an emphasis on linking risks of Valley fever to personal impacts on target populations in Central California (158). With Californians moving to rural and arid environments, land development will increase suspended dust there, and in newly developed, coccidioidomycosis-endemic areas, Valley fever incidence is higher for both construction worker and residential populations engaging in outdoor activities (159). Early warning systems and vegetation management will be critical to protect these populations.

## Cross-Cutting Health Cobenefits from Mitigation and Adaptation

Health cobenefits occur when climate change policies have secondary benefits extending beyond their primary aim. Cobenefits from climate mitigation and adaptation policy represent a major opportunity to improve public health in California, while simultaneously achieving decarbonization or enhanced adaptation capacity. Cobenefits are important for several reasons. First, the policies that generate cobenefits would often be recommended for public health protection regardless of climate change [e.g., in California, increased active transport by biking or walking may elevate physical activity for people switching from automobiles, with ensuing health benefits (160)]. Second, unlike some health benefits from climate mitigation that occur far into the future, cobenefits can often be realized in the near term, which reduces the potential discounting that can diminish the net present value of benefits, e.g., reducing short-lived climate-forcing pollutants such as O_3_ can improve population health substantially in California (83). Finally, cobenefits can also have positive health equity implications for vulnerable groups and DACs, e.g., increasing urban green spaces in underserved areas can result in increases in life expectancy in Los Angeles, with the majority of the benefits occurring in Black and Latinx neighborhoods (161).

Recent studies have also projected that achieving carbon neutrality in California by 2050 could result in some 14,000 avoided deaths via associated reductions in air pollution with an annualized monetary benefit of US$215 billion, which would be concentrated in DACs (162). The equity benefits, however, likely depend on the pathway to achieving the carbon reductions, which emphasizes the importance of building equity considerations into policy design for climate mitigation and adaptation (163). These opportunities underscore how policies aimed at achieving climate mitigation should strive to maximize public health cobenefits.

## Conclusions and Synthesis

Several key findings result from our review. First, California already experiences a substantial climate-related burden of increased exposures and health risks, although this varies by type of exposure and category of health effects and population subgroup. Statewide mortality from wildfire smoke PM_2.5_, for example, has been linked to some more than 52,000 premature deaths from 2008 to 2018 (107) (Table 1). Inconsistencies in methods to report the health burden hinder quantitative comparative analyses. Studies report various categories of health outcomes at various spatial or temporal scales, ranging from the number of cases to the cause-specific morbidity or mortality burden. Extreme heat events are another leading climate exposure in California, but the existing evidence has generally focused on specific regions or heatwave events rather than an overarching estimate of heat burden. Moreover, this research suffers from a lack of systematic reporting for novel areas such as mental health and well-being. Efforts to standardize climate-health impact reporting in line with the GBD methods could strengthen comprehensive assessments such as the upcoming Fifth California Climate Change Assessment.

Second, high attribution confidence exists on the influence of climate change on several of the exposures that we reviewed in detail, including extreme heat, dust, wildfires, and extreme precipitation. While more evidence supports direct and proximal indirect effects, further research is needed to understand indirect climate and health pathways. For example, climate-affected droughts could reduce food production (8), and as mentioned earlier, the extent to which the health of populations in California will be affected will depend on various adaptation measures such as food imports from other regions. These complex indirect pathways to public health impacts nevertheless merit more research.

Third, populations in California and elsewhere can be exposed to overlapping, synergistic, or sequential climate hazards. Our review considered only a few exposures to cumulative or compounding impacts and possible interactions, as the evidence for tracking exposures and health outcomes is currently limited (30, 164). For example, extreme precipitation can intersect with additional climate hazards, such as flooding or landslides following wildfire events (165), or interactions between extreme heat and air pollution can amplify the effects of both exposures (166). DACs could face higher risks from multiple and high-magnitude climate-related exposures (167).

Fourth, adaptation measures intended for one climate hazard can produce health cobenefits through reducing other climate exposures. Increased vegetation, for example, could reduce the urban heat island effect and provide multiple benefits, including mitigating flood risk during extreme precipitation events, improving water purification and storage, providing wildlife habitat and recreational opportunities, and increasing life expectancy in DACs (168). One challenge in evaluating adaptation solutions lies in the uncertainties about future environmental conditions due to climate change. For instance, with extended droughts that prompt water restrictions or price increases, the existing green space available in cities could be diminished, which would potentially impact the effectiveness of green space adaptation measures (169). Emphasis can also be placed on transformative adaptation solutions aimed at addressing underlying drivers of vulnerability to climate change through innovative, systems-level change (170–172).

Fifth, our review considers climate exposures associated with adverse health effects, but some health benefits are possible as well. Climate change has largely caused increases in mean and extreme temperatures (173). The frequency and intensity of cold extremes, linked to numerous adverse health effects (174), have tended to decline across the United States, which could have health benefits, but high uncertainty exists. Accelerated Arctic warming (“Arctic Amplification”) may also contribute to these observed changes in temperature extremes. For example, southern penetration of Arctic air to the midlatitudes, including Northern California, may have produced some of the extremely cold “polar vortex” events observed in recent years (175). The connection between climate change and these polar vortex events, however, remains uncertain and requires additional research (176). Milder average winter temperatures could result in reduced risks of extreme cold, but heightened variability could also result in more extreme cold events and associated adverse health effects.

Sixth, research evidence in general, and in California in particular, lacks investigations on the long-term health effects of single or multiple exposures. For example, no studies have examined the long-term health effects of wildfire smoke on major outcomes such as cardiovascular, respiratory, and all-cause mortality. Ample evidence points to undifferentiated PM_2.5_ effects on these outcomes (177), but we lack knowledge on the specific impacts of repeated wildfire exposures on these and other important outcomes, such as asthma onset or atherosclerosis progression. Consequently, assessments of potential health impacts are unlikely to capture the full magnitude of health impacts from climate-related exposures.

Finally, California is faced with an urgent need to protect its most vulnerable populations (178). Adverse public health impacts associated with climate change disproportionately affect vulnerable populations and DACs. Low-income and racially-minoritized populations face existing disproportionate energy burdens in California, exacerbated by building inefficiencies (179); this highlights the importance of federal and state programs designed to support vulnerable populations with the uptake of health-protective adaptive technologies such as AC and weatherization (34). Overall, risks from differential exposures, higher rates of preexisting health conditions, and inadequate access to adaptation measures can result in higher cumulative impacts on DAC populations. Adaptation solutions targeting vulnerable populations and DACs require information on the current and projected health burden, building on existing statewide mapping of climate-related exposures, underlying vulnerabilities, and measures of adaptive capacity (180).

While certain populations are more vulnerable, all Californians face numerous serious threats to their health and well-being from climate change. Ongoing initiatives ranging from leveraging local community knowledge to regional or California-wide efforts can expand the cobenefits of mitigation and adaptation measures, presenting substantial opportunities to improve population health and reduce additional climate-related exposures.

## Supplementary Material

Appendix 01 (PDF)

## Data Availability

There are no data underlying this work.
