# Supplementary material for: Climate change and public health in California: A structured review of exposures, vulnerable populations, and adaptation measures
Source: Proc Natl Acad Sci U S A. 2024 Jul 29;121(32):e2310081121. doi: 10.1073/pnas.2310081121 (PMC11317598; doi:10.1073/pnas.2310081121)
Supplement: Supplementary file 1 — Appendix 01 (PDF) [file pnas.2310081121.sapp.pdf]

PNAS

Supporting Information for

Climate Change and Public Health in California: A Structured Review of Exposures, Vulnerable Populations, and Adaptation Measures

Michael Jerrett[1*], Rachel Connolly[1], Diane A. Garcia-Gonzales[1], Claire Bekker[1], Jenny T. Nguyen[1], Jason Su[2], Yang Li[3], and Miriam E. Marlier[1]

[1] Department of Environmental Health Sciences, Fielding School of Public Health, University of California Los Angeles, Los Angeles, California, 90095
[2] Department of Environmental Health Sciences, School of Public Health, University of California Berkeley, Berkeley, California, 94720
[3] Department of Environmental Science, Baylor University, Waco, Texas 76798

*Corresponding Author: Michael Jerrett
Email: mjerrett@ucla.edu

This PDF file includes:

Supporting Text
Figures S1 to S2
SI References

Key Climate and Health Pathways in California

Extreme Heat

Attribution Certainty. Human influence has unequivocally impacted and warmed the atmosphere, oceans, and lands. The Fifth National Climate Assessment assigns high confidence to the increase in frequency and severity of heatwaves in the western U.S. (1). Over the last twenty years, more than 150,000 deaths from heat waves have been recorded across the globe (2, 3). Within California, various models have attributed anthropogenic emissions to increased likelihood of extreme heat, particularly in the Los Angeles and Central Valley regions (2, 4).

Timing of Effects. Across the state, annual mean temperatures have increased more than 3°F since the beginning of the 20th century (5). Southern coastal areas of the state (referred to as South Coast Drainage) have, on average, the most significant warming trend (1896 – 2015) compared to other regions (6). Compared to a 1961-1990 baseline, statewide annual average maximum temperatures could increase 4.4°F to 5.5°F by mid-century under medium and high emissions scenarios (Representative Concentration Pathway [RCP] 4.5 and 8.5, respectively) (7), and by 8°F above baseline by the end of the century (8). The frequency and intensity of heat waves are also predicted to increase by mid-century across California, including up to an additional 4-9 days in the Central Valley (9). Extreme heat events may increase in inland areas already prone to heat waves and in coastal areas where extreme heat is less frequent (9, 10). Coastal areas may experience a larger health burden because they have much lower penetration of air conditioning (AC) (11).

Extreme Precipitation

Attribution Certainty. The Fifth National Climate Assessment assigns high confidence to the increase in extreme precipitation in the U.S. Southwest (12). The association between climate change and increased precipitation extremes is more certain than changes in annual precipitation totals (13). Most heavy precipitation events in California are linked to atmospheric rivers (AR) (14), which are concentrated bands of water vapor (15). AR events, such as the heavy precipitation that caused the Oroville Dam crisis in 2017 and led to the evacuation of some 180,000 people, are enhanced to varying degrees by climate change (16). In addition to higher precipitation totals during individual events, as the climate warms, more precipitation will fall as rain rather than snow (17). Subsequent connections to flood risk are more uncertain than precipitation changes due to multiple factors, including the intensity of extreme precipitation events, soil moisture dynamics, and snowmelt patterns (18).

Timing. California currently experiences more compressed and severe rainy seasons than in the historical record (19). Since the mid-20th century, precipitation above the 90th percentile has increased significantly in the winter season over most of California (20). AR events are expected to intensify in the future, and there is strong model agreement that extreme precipitation events will increase in this century (21). Flood damage increases exponentially with higher AR intensity and duration (22); AR events were linked to more than 95% of insured flood losses over the past 40 years in parts of coastal and northern California. The risk of extreme floods associated with extreme storm sequences, runoff, and hydrologic outcomes has already doubled (23). The occurrence of an extreme precipitation event with associated flooding is more likely than not in the next few decades (23, 24).

Wildfires

Attribution Certainty. The Fifth National Climate Assessment assigns high confidence to the occurrence of unprecedented wildfire events in the U.S. Southwest (12). In the western U.S., earlier snowmelt, increased aridity, and rising temperatures associated with climate change have dried fuels and extended the wildfire season (25). From 1984 to 2015, half of the forest wildfire burned area in the western U.S.

was attributed to climate change — driven by anthropogenic increases in temperature and vapor pressure deficit which significantly increased fuel aridity (26). Turco et al. (2023) found that anthropogenic climate change, not natural variability, was responsible for dramatic increases in summer burned area in California from 1971 to 2021 (27).

Timing of Effects. Climate change is already contributing to increased wildfire activity, which is expected to worsen in the future. Annual area burned in California increased fivefold from 1972-2018 (28), at increasingly high severity (29). Under a high climate change emissions scenario (RCP 8.5), mean wildfire burned area in California is expected to increase 77% by the end of the century (compared to 1961-1990 baseline) and large wildfires would occur 50% more frequently (30).

Climate Sensitive Air Pollution Sources

Wildfire

Attribution Certainty. Climate change influences smoke pollution through increased wildfire burned area and severity. The Fifth National Climate Assessment assigns high confidence to both the increase in more frequent and severe fires that will worsen air quality as a result of climate change, as well as the risk of exposures to smoke from wildfires growing with increased heat and aridity in some regions across the U.S. (31) (no specific confidence level was quantified for the Southwest region with respect to climate-attributable air pollution impacts from wildfire). By simulating different scenarios for climate change, population growth, and development in California, Hurteau et al. (2014) reported that climate change was the largest driving force behind projected increases in wildfire $PM_{2.5}$ emissions. Although development and the expansion of the wildland urban interface have also contributed to increased wildfire risk (32), the greatest increases in wildfire emissions in California are expected to occur in parts of northern California under federal management and unavailable for development (33).

Timing of Effects. Wildfire emissions in California are projected to increase 19 -101% above the baseline period (1961-1990) by 2100 depending on the climate change and land development scenarios (33). Other studies focused on the western U.S. project that the trend in increasing wildfire $PM_{2.5}$ emissions will continue, particularly highlighting increases in maximum wildfire-specific $PM_{2.5}$ levels (34, 35).

Landscape Dust

Attribution Certainty. The Fifth National Climate Assessment assigns high confidence to the risk of exposures to airborne dust growing with increased heat and aridity in some regions across in the U.S. (31) (no specific confidence level was quantified for the Southwest region with respect to climate-attributable air pollution impacts from dust). Trend analysis indicates an increase in springtime dust concentration and an earlier onset of the dust season over the past several decades (36). Climate models predict with high confidence a warmer and drier environment in the southwestern U.S. through the 21st century (37). This would bring more frequent and severe drought (38–40). Such conditions can modify vegetative cover and influence dust mobilization.

Timing of Effects. Increased dust pollution due to climate change is likely already happening, such as intensified dust storm activity (41), and will continue in the future. Achakulwisut et al. (2018) identified the Standardized Precipitation-Evapotranspiration Index as a useful indicator of present-day dust variability, and predicted increases of 26 - 46% in fine dust concentrations over the southwestern U.S. in spring by 2100 (42). In contrast, Pu and Ginoux (2017) found that the frequency of extreme dust days decreased slightly in spring within this region due to the reduced extent of bare land under 21st century climate change, highlighting the complex relationships between climate change and land use (43).

Aeroallergens

Attribution Certainty. The Fifth National Climate Assessment assigns high confidence to climate change-attributable existing and future increases in pollen concentrations, and lengthened pollen seasons, in some regions across in the U.S. (31). However, we recognize that there remains some uncertainty in the evidence for California in terms of this pathway, as discussed in this section. Thus, attribution certainty is assigned as medium for pollen and aeroallergen exposure in California. Substantial evidence links pollen to allergic airway diseases such as rhinitis, which occurs when the body mistakenly mounts strong immune defenses to an allergen that it perceives as a threat (44). Allergies can also contribute to conjunctivitis, an irritation of the lining of the eye. Growing evidence shows that the length of season and abundance of pollen will increase across North America under warmer conditions, projected by likely climate change scenarios (45). Other aeroallergens, such as molds, may also increase in abundance due to extreme weather events (46). Some recent studies from the San Francisco Bay Area, however, report a decreasing trend over 17 years (2002-2019) in pollen concentrations (46). This retrospective study found positive associations of pollen concentrations with temperature and precipitation, but an overall decreasing trend across some 20 species. The authors conjecture that rapid urbanization may have reduced vegetative cover and that localized temperature conditions related to land use might be driving some of the reduction. As a result, while evidence concurs on the lengthening of the season for pollen and mold, the overall impact on abundance is less clear and could vary locally given the relationship between temperature, local land use, and vegetation (46).

Timing of Effects. Recent modeling studies have suggested that longer pollen seasons and increases in pollen abundance in some locations are already occurring. These trends will likely continue throughout the century in response to rising temperatures and more frequent extreme weather events (45). Pollen concentrations, however, are not uniform across space and time. These concentrations depend on multiple factors that affect the production and dispersion of pollen grains. Some of these factors are vegetation composition, land use, climate, and weather (47, 48). Therefore, pollen levels can vary considerably within a region, as well as between different regions (49). For instance, areas with more vegetation or certain plant species may have higher pollen concentrations than areas with less vegetation or different plant species. Moreover, pollen levels can change throughout the year, depending on the flowering phenology of the plants, with windblown pollinators emitting greater quantities of pollen than those species that rely on insects or birds to distribute pollen (50). Different plants also have different flowering seasons, which can be influenced by environmental cues such as temperature, day length, and precipitation. In general, pollen concentrations may vary on a microscale, at the local level, across regions, seasonally, and from year to year. The pollen monitoring network in California also remains inadequate to assess these finer temporal and spatial variations in pollen. With only 5 stations (50), it is virtually impossible to understand these microscale variations on an ongoing basis, let alone track what impacts increasing temperature and precipitation could have on pollen distribution. This makes identification of the overarching timing of effects somewhat less certain, with high variability due to local conditions.

Likely Population Health Burden. About 24 million Americans suffer from seasonal allergies related to pollen and mold exposure (51). In addition, some literature (1) suggests that air pollution exposure can interact synergistically with aeroallergens and increase the likelihood that a person will develop allergic sensitization. Although there are no specific estimates for California, several studies have shown that the increase in heightened sensitization could lead to the expenditure of millions of additional healthcare dollars. Current estimates suggest about $3 billion per year in medical costs nationally with some 3.1 million missed work days (44). Moreover, a significant, unquantified expense for those tending to individuals with allergies is likely substantial.

Vulnerable Populations. People with existing allergies and other respiratory illnesses, such as chronic obstructive pulmonary disease or asthma, are most sensitive to the increase in airborne pollen and mold. Aeroallergens may also exacerbate asthma symptoms (52). Further, a phenomenon known as “thunderstorm asthma epidemics” can occur when a storm contributes to aeroallergen exposure due to

complex processes of convection and downdrafts that can transform and liberate respirable pollens, usually from grasses, or less commonly, molds. If a sensitized group is then exposed to circulating respirable aeroallergens, allergic asthma epidemics can occur (53). As a result, asthmatics living in areas that experience more frequent or severe thunderstorms could be more vulnerable to effects than the general population. Though limited, some evidence suggests that pollen can act as an adjuvant by interacting with other common pollutants such as diesel exhaust to amplify health effects of the pollutant (54). Related evidence suggests that air pollution can increase the allergenicity of pollen (54). Thus, people living in areas with higher air pollution concentrations may also be at higher risk from pollen or mold exposure, although the evidence base on this issue is limited.

Adaptation Measures. Pharmaceuticals and related immunotherapies are the primary means of defense against allergic rhinitis and related airway disease. People suffering from allergies may also employ indoor air filters or air conditioning to reduce exposure. Some evidence suggests that living in areas with more green space can protect against allergic sensitization, with possible mediation by the gut microbiome in early life (55), but others suggest that green space can increase the risk of allergies (56). Thus, the evidence is not conclusive enough to recommend increasing green space as a possible adaptive measure. While evidence on aggregate green space is inconclusive, some plants produce less airborne pollen, which can reduce exposures for people suffering from allergies (57). Instituting regulations on plants that emit more pollen could require cities and individuals to plant species that emit lower levels of aeroallergens (58). Educational and subsidy programs for homeowners and renters to plant low pollen producing plants may help to reduce the overall pollen burden (57).

Infectious Diseases

<u>Arboviruses: West Nile Virus (WNV)</u>

Of the 15 arboviruses (**ar**thropod-**bo**rne) transmitted by mosquitoes, only three have caused significant human disease in California, specifically WNV, St. Louis encephalitis virus (SLEV), and western equine encephalitis virus (WEEV) (59). WNV was first discovered in the United States in 1999. By 2003, cases were detected in California. It is now the most important vector-borne disease in California (60). From 2003 to 2022, there were 7,597 WNV human cases and 345 deaths in California (61). Most cases occurred in the Central Valley and Southern California, with peak transmission occurring in the warmer months from July to October (62). Despite rising temperatures over the 2003-2022 period, no clear temporal pattern was present in the incidence of cases with considerable year-to-year variation (61). The other two arboviruses have had relatively little population impact, with no cases of WEEV detected in humans since 1986, and since its reemergence in 2015, only 42 cases of SLEV have been detected (61). Thus, we focus here on WNV given its relatively larger burden.

Attribution Certainty. The Fifth National Climate Assessment does not formally assess attribution certainty for WNV, so we do not provide a classification here, though we present a discussion of the available regional evidence. All aspects of WNV ecology can be influenced by climatic conditions. Sophisticated modeling studies of mosquito populations, including their spatiotemporal patterns, suggest that in many areas of California, future climates will lengthen the mosquito season, but also reduce populations during summer (62). Viral transmission via mosquito vectors displays substantial local variation, driven mainly by temperature and humidity patterns. Seasonal timing of precipitation and event intensity can also influence mosquito populations. The complex topography of California also accentuates localized variability in climate conditions and subsequent mosquito populations. It is uncertain whether population declines in the summer could lead to lower transmission to humans because drier conditions can also heighten interactions between vectors and their avian hosts as they crowd together around scarce supplies of water. Such local heterogeneity suggests that public health officials need to tailor control interventions for future planning (62). Thus, while it appears likely that climate change will alter mosquito populations (and potentially avian host distributions), this will likely have large local variation and could be influenced by the effectiveness of surveillance and management interventions. Though the Fifth National Climate Assessment does not make a specific confidence statement for WNV, it states that cases are projected to increase due to various factors—including climate change— in California (12).

Timing of Effects. As mentioned earlier, no clear temporal pattern is observed in the incidence of WNV, despite clear upward trends in temperature and extreme precipitation, which likely influence the range and spread of this vector-borne disease. Earlier modeling compared the 1970–1999 period to 2021–2050 and showed some moderate increases in the number of days with mosquitoes per month were likely to occur, particularly in later winter and early spring periods (62). The specific timing of such increases and whether they would in fact result in greater incidence of human disease remains uncertain.

Likely Population Health Burden. Most people (80%) infected with WNV experience no symptoms (63). Some people (20%) will suffer from fever with associated head and body aches, joint pains, gastrointestinal illness, and skin rash. A small minority (about 1 in 150) experience more serious symptoms including neuroinvasive disease (63). This is characterized by encephalitis, meningitis, acute flaccid paralysis, numbness and paralysis, and vision loss (63, 64). While concerning and worthy of control measures, the overall burden of illness of WNV will likely remain low compared to other climate-connected diseases. With about 17 deaths per year on average since its first detection in California, even a doubling of this number of deaths would not represent a major impact on a population that recorded 335,000 annual deaths in 2021 (65).

Vulnerable Populations. People over 60 are at a higher risk for experiencing severe infection (63). Other factors include a history of cardiovascular disease, chronic renal disease, hepatitis C viral infection, immunosuppression, and some genomic characteristics (63, 64). Occupationally, outdoor workers are at a higher risk of contracting the virus due to their increased exposure to mosquito bites. Laboratory workers handling blood of infected patients also face a higher risk of contracting the virus (66). People living near stagnant or slow-moving water with dense vegetation also appear to be at a higher risk of contracting the virus (64).

Adaptation Measures. Mosquito population control is the primary intervention for reducing the spread of WNV. This can take many forms, including better stormwater management and education campaigns targeted at home and business owners to reduce standing pools of water on their properties. Vegetation thinning and management can also be used for larval control as vegetated areas provide food and shelter for the larvae (59). Pesticides are also used to reduce larval and adult mosquito populations.

California has an advanced surveillance system that includes monitoring of mosquito abundance, collection, and analysis of infected mosquitoes, and tracking of potentially infected dead birds (59). California also tracks immune response to the virus in chickens, which are strategically located in areas with high mosquito abundance or with a history of higher WNV infections. While human disease surveillance is also part of the program, this is not considered to be a sensitive indicator due to the high prevalence of asymptomatic or mild cases. Recent studies, however, have reported strong correlations between human cases and future outbreaks (67). Broader landscape interventions are also possible. For example, innovative and cooperative approaches to restoring wetlands in the Pacific Northwest have not increased mosquito habitat or abundance (68). Several individual adaptations are available for people spending time outdoors or outdoor workers. Use of approved insecticides and repellents can reduce biting by mosquitoes. Wearing long-sleeved shirts and long pants also reduces exposure, especially when clothing is treated with 0.5% permethrin. Use of screens or air conditioners can also reduce the potential risk of being bitten by mosquitoes (69).

Air Pollution Formation

Ozone (O_3) and particulate matter (PM) are the two major air pollutants of concern because as criteria pollutants they are both regulated, and much of the air pollution-health effects research has focused on these pollutants.

Attribution Certainty. The Fifth National Climate Assessment does not formally assess attribution certainty for air pollution formation, so we do not provide a classification here, though we present a discussion of the available evidence. Tropospheric O_3 is formed through chemical reactions in the atmosphere in the presence of nitrogen oxides (NOx), volatile organic compounds (VOCs), and sunlight

(70). Anthropogenic sources of NOx and VOCs that contribute to O_3 formation include vehicle-tailpipe emissions, fossil fuel combustion and energy, and evaporation of gasoline and fuels (70). Secondary organic aerosols (SOAs) can also be formed through oxidation of VOCs (70) and have implications for worsened health impacts from highly oxidized aerosols (71). The effects of climate change on O_3 formation are inconsistent across studies, although the balance of evidence suggests that surface O_3 concentrations, peak O_3 events, or both, will likely increase as a result of climate change (72–74). Increased temperature from global warming is a crucial factor impacting O_3 concentrations, especially in areas that are highly polluted and populated (73–75). Temperature can also influence O_3 precursors, where isoprene emissions, a type of biogenic VOC (BVOC), increase in higher temperatures and NOx concentrations increase in lower temperatures. However, biogenic emissions are not always positively correlated with temperature due to the vegetation defense mechanism (76, 77). High O_3 concentrations were strongly correlated with temperature in polluted regions, and models found larger increases in O_3 due to climate change in areas that already had high O_3 levels, such as urban areas (75), which can be due to elevated levels of O_3 precursors (78). Increased humidity, BVOCs, and solar radiation can also increase O_3 concentrations, though model perturbation studies found the effects from solar radiation to be weaker (73, 75). With respect to humidity however, some studies did report declines in baseline or background O_3 levels due to higher water vapor in areas with low nitrogen oxide (NOx) concentrations, which can scavenge O_3 and reduce its lifetime (74, 75). The supply of NOx in the atmosphere is important, as areas that are limited by the amount of available NOx are NOx-limited regimes, while areas with higher NOx can be NOx-saturated or VOC-limited regimes (79). Under different regimes, the rate of O_3 formation is driven by different precursors, and thus changes in NOx and VOCs can influence O_3 differently (80). Increased cloud cover could also contribute to decreases in O_3, by blocking sunlight and reducing O_3 photochemical activity (81). Additionally, uncertainties exist with projecting high O_3 events due to variability of occurrence, and high variation can exist across different areas (74).

PM consists of primary aerosols emitted from direct sources or secondary aerosols from chemical reactions in the atmosphere (82). The effects of climate change on secondary PM are even more uncertain than O_3, with inconsistencies across studies. Both precipitation and boundary layer mixing depth impact PM formation, but projections of these two factors can be inaccurate, with unclear effects of climate change on mixing layer depth (75). Some studies have reported declines in secondary PM due to climate change-induced increases in precipitation and wet deposition, but effects are extremely variable across different areas due to differences in regional precipitation (74, 75). Higher temperatures could lead to reductions in total PM and SOAs as a result of increased volatilization of hydrocarbons, while higher humidity could marginally increase total PM and SOAs (83). Areas with high pollution could see increases in PM concentrations with increasing stagnation (75). Dust could also potentially increase secondary PM concentrations by serving as a surface for various chemical reactions (74). Additional factors that contribute to the uncertainties of the effects on PM include variability in the components comprising PM and the need for a more comprehensive understanding of the reaction chemistry with PM (72). In California, $PM_{2.5}$ concentrations were projected to decline in the southern San Joaquin Valley (SJV) and along the coast under a "business as usual" scenario (73). Secondary PM was projected to increase in certain areas within California, including the northern SJV, the southern Sacramento Valley Air Basin, the eastern South Coast Air Basin, and southeastern California (73).

Timing of Effects. As with attribution certainty, the timing of effects is also uncertain. In California, research has projected increases in O_3 in Los Angeles and the Sacramento Valley by 2090 (84), though other western US studies found insignificant changes (85). Across the US, O_3 was projected to increase by mid-century (2030 and 2050) and late-century (2080) (74, 86, 87), though effects varied across different regions within the country (88). Other studies found reductions in O_3 concentrations in the northern hemisphere between 2000 to 2030 (89), and reductions in PM in parts of California and the US by the 2050s (73, 89, 90).

Likely Population Health Burden. Climate change-induced changes in O_3 and PM formation could potentially have significant impacts on population health, though evidence for these health effects is inconsistent due to low attribution certainty of climate change impacts on air pollution formation. Additionally, evidence for health burdens on populations within California is limited. Studies that did observe projected increases in O_3 due to changes in temperature and climate also estimated projected

increases in mortality linked to O_3 by mid-century (2030 and 2050) and late-century (2090s) in California and several U.S. cities (91, 92). Mitigating climate change under two stabilization scenarios resulted in up to 22,000 avoided deaths by 2050 and up to 95,000 avoided deaths by 2100 from reduced O_3 and $PM_{2.5}$ in the U.S. (93). This analysis did not consider emissions reductions, which could imply that this study is underestimating potential health benefits. In California, air pollution exposure from emissions has been associated with mortality burdens of up to 26,700 deaths from $PM_{2.5}$ and up to 13,700 deaths from O_3 annually (specifically for 2012) (94), underscoring the potential for large health burdens from climate change-induced air pollution.

Vulnerable Populations. Few studies have examined vulnerability to future changes in O_3 and PM formation attributed to climate change. Certain populations are currently more vulnerable to air pollution, namely the elderly, racially-minoritized populations, individuals with pre-existing conditions, individuals with lower socioeconomic status, and women (95–97). Additional protective measures should be taken to prevent the potential for disproportionate impacts from climate change-induced air pollution on these populations.

Adaptation Measures. As air pollution formation could potentially increase with climate change, joint regulations that target both climate change and air pollution and incorporate input from relevant stakeholders or impacted communities could be viable solutions (72). These policies may target specific sectors of interest, such as urban planning, and could yield various health benefits from mitigated climate change and air pollution effects (72). Setting additional air pollution standards, particularly on methane and black carbon, could help reduce warming from climate change and lower background O_3 and regional PM levels (74, 98). Implementation of green infrastructure, including green roofs and green walls, could lessen air pollution concentrations, especially for carbon dioxide and O_3, and allow simultaneous improvements for both air quality and climate change (99).

Valley Fever

Attribution Certainty. The Fifth National Climate Assessment does not formally assess attribution certainty for Valley Fever, so we do not provide a classification here, though we present a discussion of the available regional evidence. *Coccidioides* reproduces rapidly during winter with moderate rainfall and mild temperature and then disperses easily during hot and dry summer conditions (100). When the soil is dry, spores of *Coccidiodes* can be picked up by wind and carried by dust particles, allowing them to be inhaled by humans (101). *Coccidiodes* spores are about 2 μm to 5 μm in length, falling within both fine and coarse dust particle sizes (102). *Coccidiodes* fungus can outlive other organisms and become dormant under drought conditions; the fungus can then be reactivated when ideal conditions (e.g., precipitation) return; this life cycle is followed by the release of infection fragments when the environment is dry and hot again and the contaminated soil is disturbed (103–105). Head et al. (2022) found that Valley fever incidence in arid counties is more sensitive to precipitation fluctuations; drought years followed by wet winters increases Valley fever incidence in California, while the incidence in cool and wet counties, such as coastal regions, is more sensitive to temperature changes (106). These regional differences create a heterogeneous spread across California. Though the Fifth National Climate Assessment does not make a specific confidence statement regarding attribution for Valley fever, it states that the incidence has increased, and is expected to continue to increase, across the Southwest region (12).

Timing of Effects. Valley fever became more prevalent in recent decades and is projected to further expand north into the dry western U.S. (135, 136). Pearson et al. (2019) analyzed the threat of Valley fever to the general population in California due to climate change, land use, and population movement and found that coccidioidomycosis incidence could rise in the near future (particularly in Southern California) due to increasing drought, aridity, and dust storms attributed to climate change. California is expected to face increasingly dry conditions, which could allow the dominant Coccidioides species (*C. immitis*) to outnumber its microbial competitors (109).

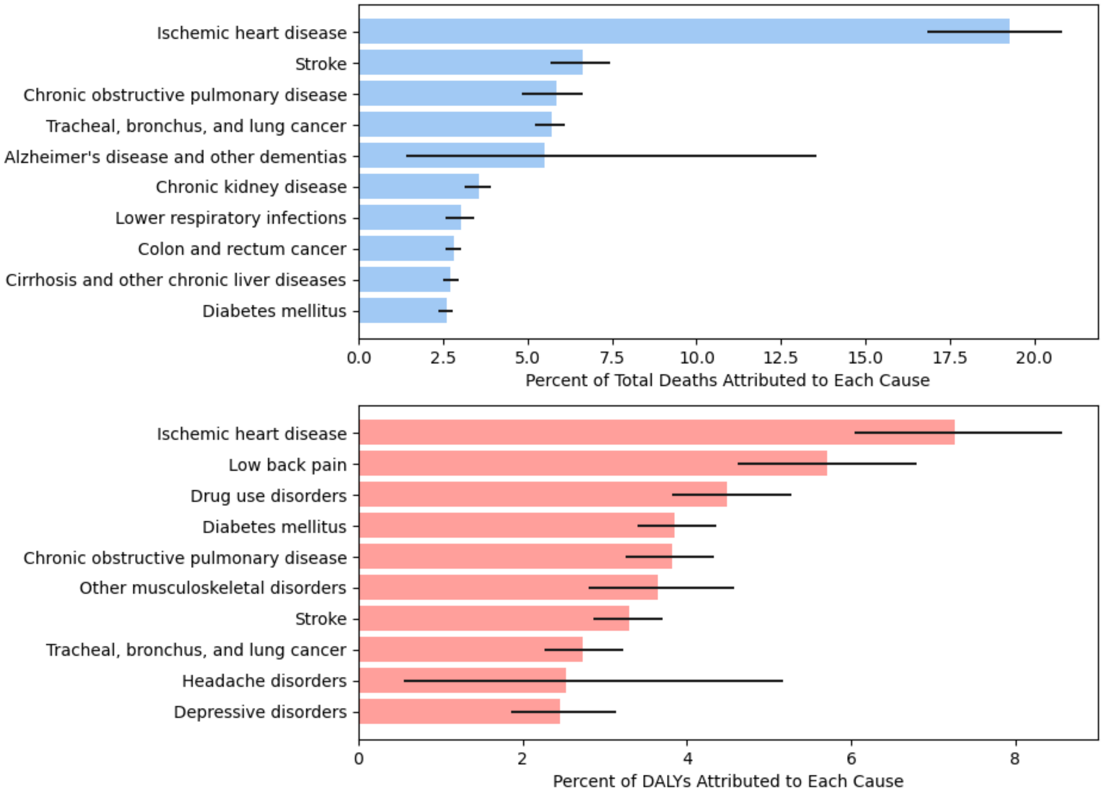

Figure S1. Leading causes of death and disability for California in 2019 with error bars representing 95% confidence intervals for each cause (110).

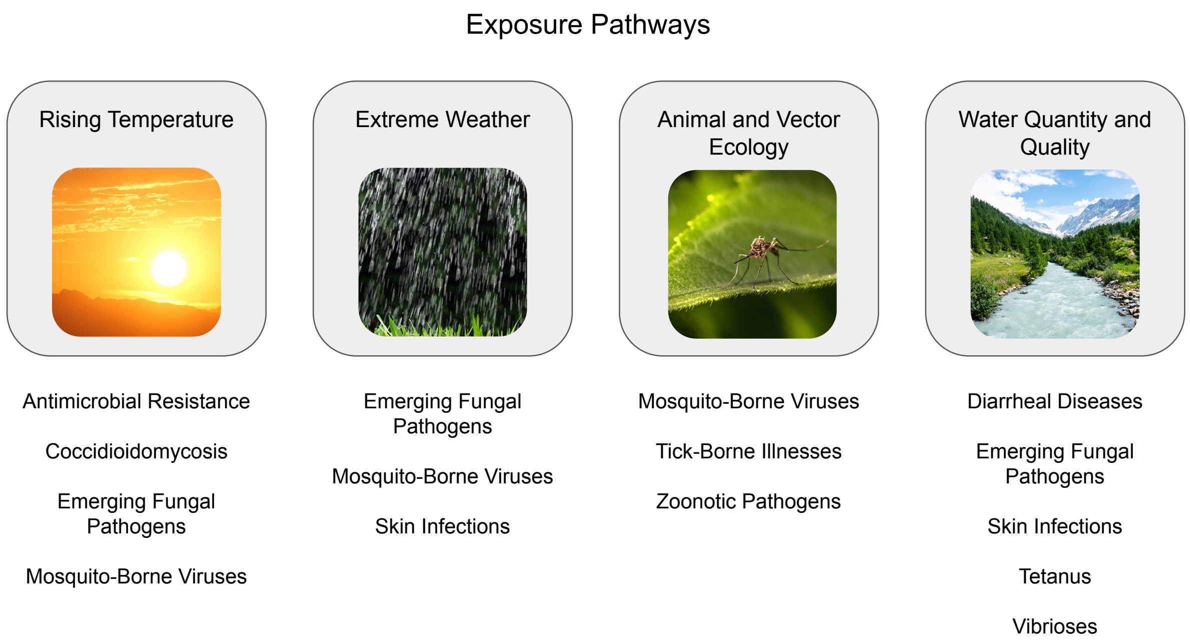

Figure S2. Climate change exposure pathways and infectious disease risks. Adapted from Edelson et al. (2022) (111).

SI References

1. K. Marvel, *et al.*, "Chapter 2. Climate trends. In: Fifth National Climate Assessment." (U.S. Global Change Research Program, Washington, DC, 2023).
2. B. Clarke, F. Otto, R. Stuart-Smith, L. Harrington, Extreme weather impacts of climate change: an attribution perspective. *Environ. Res.: Climate* **1**, 012001 (2022).
3. Intergovernmental Panel On Climate Change, *Climate Change 2021 – The Physical Science Basis: Working Group I Contribution to the Sixth Assessment Report of the Intergovernmental Panel on Climate Change*, 1st Ed. (Cambridge University Press, 2023).
4. O. Mazdiyasni, M. Sadegh, F. Chiang, A. AghaKouchak, Heat wave Intensity Duration Frequency Curve: A Multivariate Approach for Hazard and Attribution Analysis. *Sci Rep* **9**, 14117 (2019).
5. R. Frankson, *et al.*, "State Climate Summaries for the United States 2022. NOAA Technical Report NESDIS 150." (NOAA NESDIS, 2022).
6. M. He, M. Gautam, Variability and Trends in Precipitation, Temperature and Drought Indices in the State of California. *Hydrology* **3**, 14 (2016).
7. Cal-Adapt. (2018). Available at: https://cal-adapt.org/tools/extreme-heat/ [Accessed 8 December 2023].
8. L. Bedsworth, D. Cayan, G. Franco, L. Fisher, S. Ziaja, "California's Fourth Climate Change Assessment: Statewide Summary Report" (California Governor's Office of Planning and Research, Scripps Institution of Oceanography, California Energy Commission, California Public Utilities Commission, 2018).
9. P. Vahmani, A. D. Jones, C. M. Patricola, Interacting implications of climate change, population dynamics, and urban heat mitigation for future exposure to heat extremes. *Environ. Res. Lett.* **14**, 084051 (2019).
10. A. Gershunov, K. Guirguis, California heat waves in the present and future. *Geophys. Res. Lett.* **39** (2012).
11. J. Cheng, *et al.*, Cardiorespiratory effects of heatwaves: A systematic review and meta-analysis of global epidemiological evidence. *Environmental Research* **177**, 108610 (2019).
12. D. D. White, *et al.*, "Chapter 28: Southwest. In: Fifth National Climate Assessment" (U.S. Global Change Research Program, Washington, DC, 2023).
13. N. Berg, A. Hall, Increased Interannual Precipitation Extremes over California under Climate Change. *Journal of Climate* **28**, 6324–6334 (2015).
14. A. Gershunov, *et al.*, Precipitation regime change in Western North America: The role of Atmospheric Rivers. *Scientific Reports* **9**, 9944 (2019).
15. F. M. Ralph, M. D. Dettinger, M. M. Cairns, T. J. Galarneau, J. Eylander, Defining "Atmospheric River": How the Glossary of Meteorology Helped Resolve a Debate. *Bulletin of the American Meteorological Society* **99**, 837–839 (2018).
16. A. C. Michaelis, *et al.*, Atmospheric River Precipitation Enhanced by Climate Change: A Case Study of the Storm That Contributed to California's Oroville Dam Crisis. *Earth's Future* **10**, e2021EF002537 (2022).
17. X. Huang, D. L. Swain, A. D. Hall, Future precipitation increase from very high resolution ensemble downscaling of extreme atmospheric river storms in California. *Science Advances* **6**, eaba1323 (2020).
18. A. Sharma, C. Wasko, D. P. Lettenmaier, If Precipitation Extremes Are Increasing, Why Aren't Floods? *Water Resources Research* **54**, 8545–8551 (2018).
19. J. Luković, J. C. H. Chiang, D. Blagojević, A. Sekulić, A Later Onset of the Rainy Season in California. *Geophysical Research Letters* **48** (2021).

20. D. Zamora-Reyes, B. Black, V. Trouet, Enhanced winter, spring, and summer hydroclimate variability across California from 1940 to 2019. *International Journal of Climatology* **42**, 4940–4952 (2022).

21. S. D. Polade, A. Gershunov, D. R. Cayan, M. D. Dettinger, D. W. Pierce, Precipitation in a warming world: Assessing projected hydro-climate changes in California and other Mediterranean climate regions. *Scientific Reports* **7**, 10783 (2017).

22. T. W. Corringham, F. M. Ralph, A. Gershunov, D. R. Cayan, C. A. Talbot, Atmospheric rivers drive flood damages in the western United States. *Sci. Adv.* **5**, eaax4631 (2019).

23. X. Huang, D. L. Swain, Climate change is increasing the risk of a California megaflood. *Science Advances* **8**, eabq0995 (2022).

24. D. L. Swain, B. Langenbrunner, J. D. Neelin, A. Hall, Increasing precipitation volatility in twenty-first-century California. *Nature Climate Change* **8**, 427–433 (2018).

25. A. L. Westerling, Increasing western US forest wildfire activity: sensitivity to changes in the timing of spring. *Philosophical Transactions of the Royal Society B: Biological Sciences* **371**, 20150178 (2016).

26. J. T. Abatzoglou, A. P. Williams, Impact of anthropogenic climate change on wildfire across western US forests. *PNAS* **113**, 11770–11775 (2016).

27. M. Turco, *et al.*, Anthropogenic climate change impacts exacerbate summer forest fires in California. *Proceedings of the National Academy of Sciences* **120**, e2213815120 (2023).

28. A. P. Williams, *et al.*, Observed Impacts of Anthropogenic Climate Change on Wildfire in California. *Earth's Future* **7**, 892–910 (2019).

29. S. A. Parks, J. T. Abatzoglou, Warmer and Drier Fire Seasons Contribute to Increases in Area Burned at High Severity in Western US Forests From 1985 to 2017. *Geophysical Research Letters* **47**, e2020GL089858 (2020).

30. A. L. Westerling, Wildfire simulations for California's Fourth Climate Change Assessment: Projecting changes in extreme wildfire events with a warming climate (California's Fourth Climate Change Assessment, California Energy Commission). (2018).

31. J. J. West, *et al.*, "Chapter 14 : Air Quality. Fifth National Climate Assessment" (U.S. Global Change Research Program, Washington DC, 2023).

32. V. C. Radeloff, *et al.*, Rapid growth of the US wildland-urban interface raises wildfire risk. *Proc. Natl. Acad. Sci. U.S.A.* **115**, 3314–3319 (2018).

33. M. D. Hurteau, A. L. Westerling, C. Wiedinmyer, B. P. Bryant, Projected Effects of Climate and Development on California Wildfire Emissions through 2100. *Environ. Sci. Technol.* **48**, 2298–2304 (2014).

34. J. C. Liu, *et al.*, Particulate air pollution from wildfires in the Western US under climate change. *Climatic Change* **138**, 655–666 (2016).

35. X. Yue, L. J. Mickley, J. A. Logan, J. O. Kaplan, Ensemble projections of wildfire activity and carbonaceous aerosol concentrations over the western United States in the mid-21st century. *Atmospheric Environment* **77**, 767–780 (2013).

36. J. Hand, *et al.*, Earlier onset of the spring fine dust season in the southwestern United States. *Geophysical Research Letters* **43**, 4001–4009 (2016).

37. Intergovernmental Panel On Climate Change (Ipcc), *Climate Change 2022 – Impacts, Adaptation and Vulnerability: Working Group II Contribution to the Sixth Assessment Report of the Intergovernmental Panel on Climate Change*, 1st Ed. (Cambridge University Press, 2023).

38. A. F. Prein, G. J. Holland, R. M. Rasmussen, M. P. Clark, M. R. Tye, Running dry: The US Southwest's drift into a drier climate state. *Geophysical Research Letters* **43**, 1272–1279 (2016).

39. R. Seager, G. A. Vecchi, Greenhouse warming and the 21st century hydroclimate of southwestern North America. *Proc Natl Acad Sci U S A* **107**, 21277–82 (2010).

40. A. P. Williams, *et al.*, Large contribution from anthropogenic warming to an emerging North American megadrought. *Science* **368**, 314–318 (2020).

41. D. Q. Tong, J. X. L. Wang, T. E. Gill, H. Lei, B. Wang, Intensified dust storm activity and Valley fever infection in the southwestern United States. *Geophys Res Lett* **44**, 4304–4312 (2017).

42. P. Achakulwisut, L. Mickley, S. Anenberg, Drought-sensitivity of fine dust in the US Southwest: Implications for air quality and public health under future climate change. *Environmental Research Letters* **13**, 054025 (2018).

43. B. Pu, P. Ginoux, Projection of American dustiness in the late 21(st) century due to climate change. *Sci Rep* **7**, 5553 (2017).

44. Allergens and Pollen | CDC. (2020). Available at: https://www.cdc.gov/climateandhealth/effects/allergen.htm [Accessed 4 December 2023].

45. W. R. L. Anderegg, *et al.*, Anthropogenic climate change is worsening North American pollen seasons. *Proceedings of the National Academy of Sciences* **118**, e2013284118 (2021).

46. B. Paudel, *et al.*, Increased duration of pollen and mold exposure are linked to climate change. *Sci Rep* **11**, 12816 (2021).

47. B. Addison-Smith, *et al.*, Medium-Term Increases in Ambient Grass Pollen Between 1994-1999 and 2016-2020 in a Subtropical Climate Zone. *Frontiers in Allergy* **2** (2021).

48. P. J. Schramm, *et al.*, A systematic review of the effects of temperature and precipitation on pollen concentrations and season timing, and implications for human health. *Int J Biometeorol* **65**, 1615–1628 (2021).

49. A. Kenđel, B. Zimmermann, Chemical Analysis of Pollen by FT-Raman and FTIR Spectroscopies. *Front Plant Sci* **11**, 352 (2020).

50. S. Jochner, A. Menzel, R. Gehrig, B. Clot, Decrease or increase? Temporal changes in pollen concentrations assessed by Bayesian statistics. *Aerobiologia* **35** (2019).

51. Allergy Statistics in the US | Allergy & Asthma Network. Available at: https://allergyasthmanetwork.org/allergies/allergy-statistics/ [Accessed 1 December 2023].

52. R. E. Dales, S. Cakmak, S. Judek, F. Coates, Tree Pollen and Hospitalization for Asthma in Urban Canada. *International Archives of Allergy and Immunology* **146**, 241–247 (2008).

53. A. Kevat, Thunderstorm Asthma: Looking Back and Looking Forward. *J Asthma Allergy* **13**, 293–299 (2020).

54. F. Sedghy, A.-R. Varasteh, M. Sankian, M. Moghadam, Interaction Between Air Pollutants and Pollen Grains: The Role on the Rising Trend in Allergy. *Rep Biochem Mol Biol* **6**, 219–224 (2018).

55. V. Buchholz, *et al.*, Natural Green Spaces, Sensitization to Allergens, and the Role of Gut Microbiota during Infancy. *mSystems* **8**, e01190-22 (2023).

56. L. Ruokolainen, Green living environment protects against allergy, or does it? *European Respiratory Journal* **49** (2017).

57. Welcome to Allergy Free Gardening! Available at: http://www.allergyfree-gardening.com/ [Accessed 7 December 2023].

58. B. Sawers, Regulating Pollen. (2014).

59. California Department of Public Health, Mosquito & Vector Control Association of California, University of California, CALIFORNIA MOSQUITO-BORNE VIRUS SURVEILLANCE & RESPONSE PLAN. (2023).

60. Westnile.ca.gov | California West Nile Virus Website. (2023). Available at: https://westnile.ca.gov/ [Accessed 1 December 2023].

61. C. E. P. A. Office of Environmental Health Hazard Assessment, Indicators of Climate Change in California. (2018).

62. C. W. Morin, A. C. Comrie, Regional and seasonal response of a West Nile virus vector to climate change. *Proceedings of the National Academy of Sciences* **110**, 15620–15625 (2013).

63. Symptoms, Diagnosis, & Treatment | West Nile Virus | CDC. (2023). Available at: https://www.cdc.gov/westnile/symptoms/index.html [Accessed 1 December 2023].

64. R. R. Montgomery, K. O. Murray, Risk factors for West Nile virus Infection and Disease in Populations and Individuals. *Expert Rev Anti Infect Ther* **13**, 317–325 (2015).

65. Statewide Death Profiles - 2014-2021 Final Deaths by Year Statewide - California Health and Human Services Open Data Portal. Available at: https://data.chhs.ca.gov/dataset/statewide-death-profiles/resource/f8bb67bf-923d-4d74-8714-dc2bcf5609b7?filters=Year%3A2021%7CCause_Desc%3AAll%20causes%20(total) [Accessed 4 December 2023].

66. U.S. Department of Labor, Occupational Safety and Health Administration, Directorate of Enforcement Programs, Office of Health Enforcement, Workplace Precautions Against West Nile Virus | Occupational Safety and Health Administration. (2012). Available at: https://www.osha.gov/publications/shib082903b#:~:text=What%20occupations%20are%20at%20risk,more%20likely%20to%20be%20present [Accessed 1 December 2023].

67. M. E. Danforth, R. E. Snyder, E. T. N. Lonstrup, C. M. Barker, V. L. Kramer, Evaluation of the effectiveness of the California mosquito-borne virus surveillance & response plan, 2009–2018. *PLOS Neglected Tropical Diseases* **16**, e0010375 (2022).

68. A. Beehler, D. Markowski, M. Crowder, K. E. Iaquinto, J. Kinley, Controlling mosquitoes through innovative and collaborative wetland management practices in the Pacific Northwest. *Wetlands Ecol Manage* **30**, 975–985 (2022).

69. Prevent Mosquito Bites | Mosquitoes | CDC. (2023). Available at: https://www.cdc.gov/mosquitoes/mosquito-bites/prevent-mosquito-bites.html [Accessed 1 December 2023].

70. J. (Jim) Zhang, Y. Wei, Z. Fang, Ozone Pollution: A Major Health Hazard Worldwide. *Front Immunol* **10**, 2518 (2019).

71. F. Liu, T. Xu, N. L. Ng, H. Lu, Linking Cell Health and Reactive Oxygen Species from Secondary Organic Aerosols Exposure. *Environ. Sci. Technol.* **57**, 1039–1048 (2023).

72. P. L. Kinney, Interactions of Climate Change, Air Pollution, and Human Health. *Curr Envir Health Rpt* **5**, 179–186 (2018).

73. M. J. Kleeman, S.-H. Chen, R. A. Harley, "Climate Change Impact on Air Quality in California: Report To The California Air Resources Board" (2010).

74. A. M. Fiore, *et al.*, Global air quality and climate. *Chem. Soc. Rev.* **41**, 6663–6683 (2012).

75. D. J. Jacob, D. A. Winner, Effect of climate change on air quality. *Atmospheric Environment* **43**, 51–63 (2009).

76. E. Austin, *et al.*, Ozone Trends and their Relationship to Characteristic Weather Patterns. *J Expo Sci Environ Epidemiol* **25**, 532–542 (2015).

77. S. Dewan, A. Lakhani, Tropospheric ozone and its natural precursors impacted by climatic changes in emission and dynamics. *Frontiers in Environmental Science* **10** (2022).

78. M. L. Bell, *et al.*, Climate change, ambient ozone, and health in 50 US cities. *Climatic Change* **82**, 61–76 (2007).

79. J. Ren, F. Guo, S. Xie, Diagnosing ozone–NO_x–VOC sensitivity and revealing causes of ozone increases in China based on 2013–2021 satellite retrievals. *Atmospheric Chemistry and Physics* **22**, 15035–15047 (2022).

80. US EPA, “Final Ozone NAAQS Regulatory Impact Analysis” (2008).

81. J. P. Dawson, P. J. Adams, S. N. Pandis, Sensitivity of ozone to summertime climate in the eastern USA: A modeling case study. *Atmospheric Environment* **41**, 1494–1511 (2007).

82. California Air Resources Board, Inhalable Particulate Matter and Health (PM2.5 and PM10) | California Air Resources Board. Available at: https://ww2.arb.ca.gov/resources/inhalable-particulate-matter-and-health [Accessed 5 December 2023].

83. J. R. Horne, D. Dabdub, Impact of global climate change on ozone, particulate matter, and secondary organic aerosol concentrations in California: A model perturbation analysis. *Atmospheric Environment* **153**, 1–17 (2017).

84. H. Taha, Potential impacts of climate change on tropospheric ozone in California: A preliminary episodic modeling assessment of the Los Angeles basin and the Sacramento valley. (2001).

85. K. Murazaki, P. Hess, How does climate change contribute to surface ozone change over the United States? *Journal of Geophysical Research: Atmospheres* **111** (2006).

86. N. Fann, *et al.*, The geographic distribution and economic value of climate change-related ozone health impacts in the United States in 2030. *Journal of the Air & Waste Management Association* **65**, 570–580 (2015).

87. C. Hogrefe, *et al.*, Simulating changes in regional air pollution over the eastern United States due to changes in global and regional climate and emissions. *Journal of Geophysical Research: Atmospheres* **109** (2004).

88. F. Dentener, *et al.*, The Global Atmospheric Environment for the Next Generation. *Environ. Sci. Technol.* **40**, 3586–3594 (2006).

89. E. Tagaris, *et al.*, Impacts of global climate change and emissions on regional ozone and fine particulate matter concentrations over the United States. *Journal of Geophysical Research: Atmospheres* **112** (2007).

90. J. Avise, *et al.*, Attribution of projected changes in U.S. ozone and PM2.5 concentrations to global changes. *Atmos. Chem. Phys. Discuss* **8**, 15131–15163 (2008).

91. A. Wilson, *et al.*, Climate change impacts on projections of excess mortality at 2030 using spatially varying ozone–temperature risk surfaces. *J Expo Sci Environ Epidemiol* **27**, 118–124 (2017).

92. K. L. Ebi, G. McGregor, Climate Change, Tropospheric Ozone and Particulate Matter, and Health Impacts. *Environ Health Perspect* **116**, 1449–1455 (2008).

93. F. Garcia-Menendez, R. K. Saari, E. Monier, N. E. Selin, U.S. Air Quality and Health Benefits from Avoided Climate Change under Greenhouse Gas Mitigation. *Environ. Sci. Technol.* **49**, 7580–7588 (2015).

94. T. Wang, *et al.*, Mortality burdens in California due to air pollution attributable to local and nonlocal emissions. *Environment International* **133**, 105232 (2019).

95. L. G. Hooper, J. D. Kaufman, Ambient Air Pollution and Clinical Implications for Susceptible Populations. *Ann Am Thorac Soc* **15**, S64–S68 (2018).

96. M. L. Bell, A. Zanobetti, F. Dominici, Who is More Affected by Ozone Pollution? A Systematic Review and Meta-Analysis. *Am J Epidemiol* **180**, 15–28 (2014).

97. P. Geldsetzer, *et al.*, Disparities in air pollution attributable mortality in the US population by race/ethnicity and sociodemographic factors. *Nat Med* 1–9 (2024). https://doi.org/10.1038/s41591-024-03117-0.

98. A. M. Fiore, V. Naik, E. M. Leibensperger, Air Quality and Climate Connections. *Journal of the Air & Waste Management Association* **65**, 645–685 (2015).

99. V. Anderson, W. A. Gough, Evaluating the potential of nature-based solutions to reduce ozone, nitrogen dioxide, and carbon dioxide through a multi-type green infrastructure study in Ontario, Canada. *City and Environment Interactions* **6**, 100043 (2020).

100. M. del Rocío Reyes-Montes, *et al.*, The habitat of Coccidioides spp. and the role of animals as reservoirs and disseminators in nature. *BMC infectious diseases* **16**, 1–8 (2016).

101. M. Matlock, S. Hopfer, O. A. Ogunseitan, Communicating risk for a climate-sensitive disease: A case study of valley fever in central California. *International journal of environmental research and public health* **16**, 3254 (2019).

102. S. M. Akram, J. Koirala, "Coccidioidomycosis" in *StatPearls*, (StatPearls Publishing, 2023).

103. S. J. Coates, L. P. Fox, Disseminated coccidioidomycosis as a harbinger of climate change. *JAAD Case Reports* **4**, 424–425 (2018).

104. M. C. Fisher, G. L. Koenig, T. J. White, J. W. Taylor, Pathogenic clones versus environmentally driven population increase: analysis of an epidemic of the human fungal pathogen Coccidioides immitis. *Journal of Clinical Microbiology* **38**, 807–813 (2000).

105. M. E. Gorris, L. A. Cat, C. S. Zender, K. K. Treseder, J. T. Randerson, Coccidioidomycosis Dynamics in Relation to Climate in the Southwestern United States. *Geohealth* **2**, 6–24 (2018).

106. J. R. Head, *et al.*, Effects of precipitation, heat, and drought on incidence and expansion of coccidioidomycosis in western USA: a longitudinal surveillance study. *The Lancet Planetary Health* **6**, e793–e803 (2022).

107. M. E. Gorris, J. E. Neumann, P. L. Kinney, M. Sheahan, M. C. Sarofim, Economic valuation of coccidioidomycosis (Valley fever) projections in the United States in response to climate change. *Weather, Climate, and Society* **13**, 107–123 (2021).

108. M. E. Gorris, K. K. Treseder, C. S. Zender, J. T. Randerson, Expansion of coccidioidomycosis endemic regions in the United States in response to climate change. *GeoHealth* **3**, 308–327 (2019).

109. D. Pearson, K. Ebisu, X. Wu, R. Basu, A Review of Coccidioidomycosis in California: Exploring the Intersection of Land Use, Population Movement, and Climate Change. *Epidemiologic Reviews* **41**, 145–157 (2019).

110. Global Burden of Disease Collaborative Network, Institute for Health Metrics and Evaluation (IHME), Global Burden of Disease Study 2019, Results. Deposited 2020.

111. P. J. Edelson, *et al.*, Climate Change and the Epidemiology of Infectious Diseases in the United States. *Clinical Infectious Diseases* **76**, 950–956 (2023).
